# Supplementary material for: Towards an international research agenda for public health advocacy: Practice, preparedness and knowledge gaps
Source: PLOS Glob Public Health. 2026 Jan 23;6(1):e0005713. doi: 10.1371/journal.pgph.0005713 (PMC12829807; doi:10.1371/journal.pgph.0005713)
Supplement: S1 File — (DOCX) [file pgph.0005713.s001.docx]

## **S1 File**

## **Survey questions**

1. Do you identify as a public health advocate?

- Yes
- No

1. At present, do you work for a private or commercial organization?

- Yes
- No

1. Over the last two years, have you worked for a private or commercial organization?

- Yes
- No

1. We are seeking the opinions of individuals that identify as being involved in public health advocacy with experience working in non-government organizations, government organizations, universities/research institutes, UN agencies, or foundations of a for-profit organization.

Please select the sector where you do the most advocacy work.

- Non-government organization
- Government organization
- University/Research institute
- United Nations agency
- Foundation of a for-profit organization
- Other ________________________________________________

*Display This Question:*

*Please select the sector where you do the most advocacy work = Foundation of a for-profit organization*

1. Please specify the name of the Foundation of a for-profit organization [Optional]

________________________________________________

**DEMOGRAPHIC AND PROFESSIONAL INFORMATION**

1. Which gender do you identify as? Please select all that apply

- Woman
- Man
- Non-binary/non-conforming
- Prefer not to respond

1. Which country are you physically based in? [Drop-down menu]

________________

1. Which country is the focus of your advocacy work? [Drop-down menu]

________________

1. What geographic location does your work focus on? Please select all that apply

- Local/Community-based (e.g., local council)
- State/District/County (e.g., a state or territory in a federal system of government)
- National (e.g., a country’s government)
- Regional (e.g., European Commission; African Union; ASEAN)
- International/Global (e.g., the World Health Organization, United Nations)

1. How long have you worked in public health?

- < 2 years
- 2-5 years
- 6-10 years
- >10 years

1. Which public health issues do you focus on? Please select all that apply

- Chronic Disease (e.g., food system, nutrition, physical activity, tobacco)
- Climate Change
- Communicable Diseases (e.g., HIV/AIDS, malaria, tuberculosis)
- Injury & Violence Prevention
- Mental Health
- Sexual Health
- Maternal and Child Health
- Environmental Health
- Substance Misuse
- Vaccines
- Other, please specify: ________________________________________________

***We would now like to ask you some questions about advocacy. When we refer to advocacy, we mean any active attempts you have made to influence decision makers or the public to support or implement upstream level changes (e.g., laws, regulations, policies, and institutional practices), with the intention of improving public health and wellbeing.***

1. At present, what percentage of your time do you engage in advocacy work?

Please indicate here (sliding scale): 0----------------------------------------------------------100

1. Do you engage in advocacy on a regular basis? Please select all that apply

- Regularly (e.g., daily, weekly, fortnightly, or monthly, or as part of an ongoing campaign or political advocacy)
- Irregularly (e.g., 1-2 times/year, or only when the need for a campaign or political advocacy arises;)
- Other, please specify:______________________

1. Choosing from the options below, how often have you undertaken the following advocacy strategies on a particular issue in the past 12 months?

|  | Daily | Weekly | Fortnightly | Monthly | 1-2 times/year | Never |
| --- | --- | --- | --- | --- | --- | --- |
| Written a letter to the editor on your policy issue |  |  |  |  |  |  |
| Written an article for the general public on your policy issue (published in mainstream media or online) |  |  |  |  |  |  |
| Written a submission in response to government inquiry on your policy issue |  |  |  |  |  |  |
| Written a media release on your policy issue |  |  |  |  |  |  |
| Interviewed by the media on your policy issue |  |  |  |  |  |  |
| Worked in an advocacy coalition on your policy issue |  |  |  |  |  |  |
| Commissioned polling on public opinion of your policy issue |  |  |  |  |  |  |
| Commissioned research to inform advocacy on your policy issue |  |  |  |  |  |  |
| Met with a politician regarding your policy issue |  |  |  |  |  |  |
| Met with a ‘health-related’ civil servant/government bureaucrat regarding your policy issue |  |  |  |  |  |  |
| Met with a ‘non-health’ civil servant/government bureaucrat (e.g., Ministry of Finance, Ministry of Environment) regarding your policy issue |  |  |  |  |  |  |
| Other (e.g., protests, social media campaigns), please specify: |  |  |  |  |  |  |

*Display This Question:*

*Met with a civil/public servant in a traditionally ‘non-health’ Ministry about your policy issue (e.g., Ministry of Finance, Ministry of Environment) regarding your policy issue? = Daily to 1-2 times/year*

1. If you selected ‘Met with a ‘non-health’ civil servant/government bureaucrat about your policy issue (e.g., Ministry of Finance, Ministry of Environment), please specify which ‘non-health’ Ministry/Ministries:

1. Choosing from the options below, how well equipped are you to be an effective advocate?

|  | Very well equipped | Somewhat well equipped | Not very well equipped |
| --- | --- | --- | --- |
| Skills |  |  |  |
| Knowledge |  |  |  |
| Time |  |  |  |
| Funding |  |  |  |
| Access to networks |  |  |  |
| Other (e.g., buy in [interest) from affected populations, influence/voice, credibility). Please specify __________ |  |  |  |

Any further comments? Feel free to explain your responses

________________________________________________________________

1. Have you undertaken formal training on developing and/or implementing effective advocacy strategies?

- No
- Yes

*Display This Question:*

*If Have you undertaken formal training on developing and implementing effective advocacy strategies? = Yes*

1. Please outline the type of advocacy training you have undertaken and the organisation which conducted the training.

**________________________________________________________________**

**ADVOCACY RESEARCH NEEDS**

1. a. From your personal experience, which advocacy strategy have you used that has been most effective and why?

**________________________________________________________________**

b. Have you found that the effectiveness of your advocacy strategy differs depending on the type of organisation (e.g., local vs national) you are targeting? If yes, could you explain how?

**________________________________________________________________**

1. Are there any advocacy strategies you would like more evidence to be generated to inform practice?

**________________________________________________________________**

1. Are there any advocacy strategies you would like more evidence to be generated to inform practice?

**__________________________________________________________**

END OF SURVEY
