# Supplementary material for: Towards an international research agenda for public health advocacy: Practice, preparedness and knowledge gaps
Source: PLOS Glob Public Health. 2026 Jan 23;6(1):e0005713. doi: 10.1371/journal.pgph.0005713 (PMC12829807; doi:10.1371/journal.pgph.0005713)
Supplement: S2 File — (DOCX) [file pgph.0005713.s002.docx]

**S2 File**: Location of participants

| Country | Count | Proportion | Region | Income |
| --- | --- | --- | --- | --- |
| Australia | 54 | 35% | WPR | HI |
| Canada | 14 | 9% | AMR | HI |
| United Kingdom | 11 | 7% | EUR | HI |
| New Zealand | 7 | 5% | WPR | HI |
| Ireland | 6 | 4% | EUR | HI |
| Ghana | 5 | 3% | AFR | LMIC |
| Nigeria | 5 | 3% | AFR | LMIC |
| United States of America | 5 | 3% | AMR | HI |
| India | 4 | 3% | SEAR | LMIC |
| Mexico | 3 | 2% | AMR | UMIC |
| Qatar | 3 | 2% | EMR | HI |
| Senegal | 3 | 2% | AFR | LMIC |
| Switzerland | 3 | 2% | EUR | HI |
| Brazil | 2 | 1% | AMR | UMIC |
| Brunei Darussalam | 2 | 1% | WPR | HI |
| France | 2 | 1% | EUR | HI |
| Indonesia | 2 | 1% | SEAR | UMIC |
| South Africa | 2 | 1% | AFR | UMIC |
| Uganda | 2 | 1% | AFR | LI |
| Barbados | 2 | 1% | AMR | HI |
| Belgium | 1 | <1% | EUR | HI |
| Burkina Faso | 1 | <1% | AFR | LI |
| Fiji | 1 | <1% | WPR | UMIC |
| Jamaica | 1 | <1% | AMR | UMIC |
| Japan | 1 | <1% | WPR | HI |
| Kenya | 1 | <1% | AFR | LMIC |
| Madagascar | 1 | <1% | AFR | LI |
| Nepal | 1 | <1% | SEAR | LMIC |
| Netherlands | 1 | <1% | EUR | HI |
| Norway | 1 | <1% | EUR | HI |
| Philippines | 1 | <1% | WPR | LMIC |
| Republic of Yemen | 1 | <1% | EMR | LI |
| Rwanda | 1 | <1% | AFR | LI |
| Sri Lanka | 1 | <1% | SEAR | LMIC |
| Tanzania | 1 | <1% | AFR | LMIC |
| Zambia | 1 | <1% | AFR | LMIC |
