# Supplementary material for: Towards an international research agenda for public health advocacy: Practice, preparedness and knowledge gaps
Source: PLOS Glob Public Health. 2026 Jan 23;6(1):e0005713. doi: 10.1371/journal.pgph.0005713 (PMC12829807; doi:10.1371/journal.pgph.0005713)
Supplement: Attachment — Submitted filename: response to Reviewers (1).docx [file pgph.0005713.s003.docx]

| Reviewer #1 | Author response |
| --- | --- |
| This study investigates practices, preparedness and knowledge gaps that exists in global public health advocacy through a multi-country survey and content analysis. This is a highly relevant study, which can make a significant contribution to public health advocacy literature, and holds a great value as further research can be informed through this work. The manuscript meets the publication criteria, is technically sound, and the data support the conclusions made. Appropriate data analyses have been done. Raw data is not made publicly available mentioning potential identifying information of the participants. The manuscript has a well-organized structure, and is drafted in a standard English. | Thank you! |
| 1. Since this is a multi-country survey, it is important to mention whether the survey form was offered in multiple languages or English only. Since participants who were able to comprehend and write in English were only included in the study, it seems like the form was developed and used in English version only. If so, restricting participation to English speakers should be acknowledged as a limitation. | Great point, we have added this to our limitations section (p17)  Additionally, the survey was only available in English, and most study participants were from one country, which limited our analysis across country contexts. Consequently, we may have missed valuable insights into perceived effective advocacy strategies as well as the role of public trust and acceptability of advocacy organisations in different government systems. We encourage future research to explore these questions across multiple languages and settings to capture a broader range of perspectives. |
| 2. If the survey form developed was mobile-friendly, kindly mention it in the methods section, as it clarifies accessibility issues. | Thanks for this suggestion, we’ve added it to page 6:  The survey was designed using Qualtrics software (Version 2023) with input from all authors and comprised 21 items, including multiple-choice, close-ended, and open-ended questions (see Supplementary File 1). The survey could be completed on either a desktop computer or a mobile device. |
| 3. Authors can be more transparent regarding participant recruitment and response metrics. Specifically, it does not report the number of agencies contacted, the number of individuals invited, or the overall response rate. | We appreciate the reviewer’s suggestion to provide greater transparency about participant recruitment and response metrics. Unfortunately, it is not possible to calculate a response rate for this survey, as the recruitment approach involved an open invitation distributed through professional networks and organisations, with recipients encouraged to forward the survey to relevant colleagues (a snowball sampling approach). Consequently, the total number of individuals who received the survey invitation is unknown (inestimable). However, we have added the initial number of health agencies contacted (p6):    Participants were recruited from an initial purposive sample of 100 public health agencies in low-, middle-, and high-income countries  And we have provided further clarification in the methods (p6):  As the number of individuals who received the invitation could not be tracked, it was not possible to calculate a response rate. |
| Additionally, there is no information on attrition or incomplete responses. Including a recruitment flow diagram, similar to a CONSORT-diagram adapted for surveys, would significantly improve clarity and allow readers to better understand the sampling process and data quality. | Thanks for this suggestion. We have added the additional information in the results (p7):  Three hundred and twenty seven individuals began the survey, with 153 eligible to participate.  In your response to your attrition suggestion, we were unable to track incomplete responses due to the survey’s design and anonymity requirements. Therefore, a full attrition flow diagram could not be provided. Hower, we have added additional text in the limitations (p17) to address this and regarding the slightly fewer participants providing open text responses.  Slightly fewer participants responded to the open-text questions, which may limit the depth or representativeness of qualitative insights. Additionally, due to the survey design and the requirement for anonymity, it was not possible to accurately track incomplete responses beyond eligibility screening, and we were therefore unable to provide a full attrition flow diagram. |
| Reviewer #2 |  |
| For the introduction and context, I expected to see more comment on the diversity of advocacy approaches given differing political environments and history of advocacy in countries. In some countries it is safe to advocate with the government, in some it is not. What are the implications for supporting advocacy given this diversity of settings? | Thanks for this comment. We agree that the diversity of political environments and advocacy histories across countries is an important consideration. In the revised manuscript, we have expanded the Introduction to include this (p4/5):  While these positive examples should be celebrated, public health advocates continue to face significant barriers to achieving policy reform, globally. Advocacy approaches are shaped by the diversity of political environments and histories of engagement across countries. In some contexts, it is relatively safe to engage directly with government actors, whereas in others, political constraints or risks may limit such engagement. |
| I think the research agenda could be more robust. What about studying online training and support for advocates? Given that funding is a major issue, why not study how to make the case for advocacy funding? Also, given that interaction with policy makers can be highly affective and many advocates do this and many more want to, why not study what aspects/tactics with policy maker outreach is most impactful in different situations and settings? And how to communicate those findings to other advocates? | Thank you for these thoughtful suggestions. We agree that these are important areas for future research. In response, we have added two additional, more pragmatic questions to the research agenda based on your recommendations (p18):  - What are the most effective approaches to meeting the training needs of advocates, including through online platforms?  - How can advocates demonstrate their added value and effectively campaign for additional resources to support their work?  Regarding your suggestion about identifying which aspects or tactics of policymaker outreach are most impactful across different situations and settings, we believe this is already addressed in the fourth point of our proposed research agenda:   - What are the most effective approaches for engaging with a variety of governments and decision-makers about public health, and how do these approaches vary based on political, cultural, and institutional contexts?   We hope this clarification is helpful and appreciate your contribution to strengthening the manuscript. |
